# Precision Emergency Medicine: A Systematic Review

**DOI:** 10.7759/cureus.75068

**Published:** 2024-12-03

**Authors:** Amgad Ahmed, Mazin Mustafa

**Affiliations:** 1 Emergency Medicine, Dubai Hospital, Dubai, ARE; 2 Emergency Medicine, Zayed Military Hospital, Abu Dhabi, ARE

**Keywords:** emergency care, genomic medicine, personalized medicine, point-of-care diagnostics, precision emergency medicine, precision medicine, systematic review

## Abstract

Precision medicine, which customizes healthcare based on individual genetic, environmental, and lifestyle factors, has significantly advanced various medical fields. However, its adoption in emergency medicine remains limited despite the potential to enhance patient outcomes through more accurate diagnostics and personalized treatments. This systematic review examined current evidence on the application of precision medicine in emergency care by analyzing studies published between 2010 and 2024. Out of 218 records, 10 studies met the inclusion criteria, highlighting areas such as genomic applications, machine learning, point-of-care diagnostics, and biomarkers. The findings indicate that precision medicine can improve diagnostic accuracy and personalize patient care in emergency settings, although challenges such as time constraints, technological limitations, and the need for enhanced clinician training remain. Overcoming these barriers through interdisciplinary collaboration, investment in rapid diagnostic technologies, and comprehensive education programs is essential for effectively integrating precision medicine into emergency care. Ultimately, advancing precision emergency medicine holds promise for transforming emergency care into a more personalized and effective practice, thereby improving patient outcomes in acute situations.

## Introduction and background

Precision medicine, an approach that considers individual variability in genes, environment, and lifestyle to allow for more precise prediction and treatment, has significantly influenced various medical specialties [1]. Integrating genomic data and advanced diagnostics has led to personalized therapies, particularly in fields such as oncology and cardiology [2]. However, applying precision medicine principles in emergency medicine (EM) remains relatively underexplored.

EM is characterized by the need for rapid assessment and immediate decision-making to manage acute illnesses and injuries. Traditional EM practices often rely on generalized protocols designed for the average patient, which may not account for individual variations that can impact clinical outcomes [3]. The heterogeneous nature of emergency department (ED) presentations underscores the potential benefits of adopting precision medicine approaches to tailor interventions more effectively.

Incorporating precision medicine into emergency care, termed precision emergency medicine (PEM), holds promise for enhancing diagnostic accuracy and optimizing therapeutic strategies. By leveraging patient-specific data such as genetic profiles, biomarkers, and point-of-care diagnostics, PEM could transform emergency care from a protocol-driven practice to one that is more personalized and outcome-focused [4]. For example, pharmacogenomics could guide medication selection and dosing in the ED, reducing adverse drug reactions and improving efficacy.

Several studies have begun to explore the integration of precision medicine in emergency settings, highlighting both opportunities and challenges. Kingsmore et al. (2015) demonstrated the potential of rapid whole-genome sequencing in critically ill infants, enabling timely and precise diagnoses that can significantly alter clinical management [5]. Additionally, point-of-care ultrasound (POCUS) has been identified as a tool for delivering precision care in pediatric emergencies, allowing for individualized assessment and intervention [6].

Despite these advancements, the literature on PEM is limited, with only a handful of studies directly addressing the topic. A recent search identified only a limited number of papers specifically focused on PEM, indicating a significant gap in research [4,7]. This paucity of data underscores the need for comprehensive evaluations of how precision medicine principles can be effectively implemented in emergency care to improve patient outcomes. Moreover, challenges such as limited time for data analysis in acute settings, the need for rapid diagnostic tools, and ethical considerations related to genetic testing remain significant barriers to widespread adoption [8,9].

This systematic review aims to critically evaluate the existing literature on PEM, focusing on the limited number of studies available. Specifically, it will analyze how precision medicine principles have been applied in emergency care settings, assess preliminary impacts on clinical outcomes, and identify reported barriers and facilitators to implementation. By synthesizing findings from these studies, the review seeks to provide a foundational understanding of PEM, highlight gaps in current knowledge, and propose directions for future research to advance the field.

## Review

Methods

Study Design

This systematic review was conducted to evaluate the application of precision medicine principles in emergency care settings. The review followed the Preferred Reporting Items for Systematic Reviews and Meta-Analyses (PRISMA) guidelines to ensure a transparent and replicable methodology [10].

Search Strategy

A comprehensive literature search was conducted across multiple electronic databases, including PubMed, Embase, Cochrane Library, Scopus, and Google Scholar, covering articles published from January 2010 to October 2024 to capture the latest advancements in PEM. The search, completed in October 2024, employed a strategy that combined keywords and Medical Subject Headings (MeSH) terms related to precision and emergency medicine. Example search terms included phrases such as "precision medicine", "personalized medicine", "individualized medicine", "genomic medicine", "machine learning", and "artificial intelligence", combined with terms specific to emergency settings such as "emergency medicine", "emergency department", "acute care", and "emergency services". Additionally, the reference lists of relevant articles were manually reviewed to identify further studies that met the inclusion criteria.

Inclusion and Exclusion Criteria

The inclusion and exclusion criteria for this study were defined to ensure relevance and rigor. The inclusion criteria encompassed studies involving patients in emergency care settings where precision medicine principles, such as genomics, machine learning (ML), point-of-care diagnostics, biomarkers, or personalized therapeutic strategies, were applied. Eligible study types included original research articles, reviews, editorials, consensus statements, and meta-analyses, focusing on English-language publications from January 2010 to October 2024. The exclusion criteria ruled out studies not directly related to precision medicine in emergency care, including case reports, conference abstracts, letters to the editor, non-peer-reviewed articles, and research focused on non-emergency settings or specialties outside EM.

Study Selection

**Figure 1:**
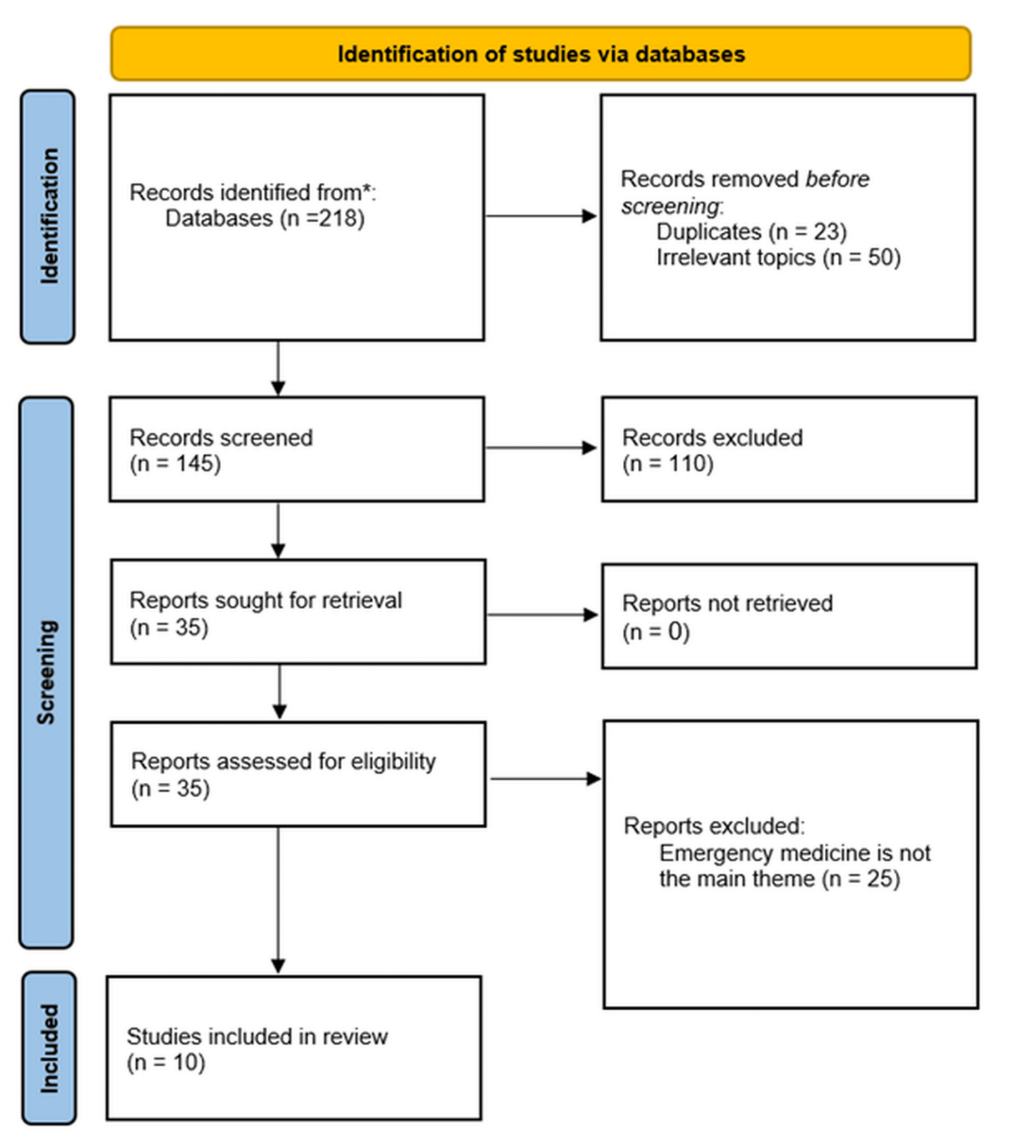
PRISMA flow diagram of the study selection process. PRISMA: Preferred Reporting Items for Systematic Reviews and Meta-Analyses

All identified articles were imported into EndNote (Clarivate, Philadelphia, Pennsylvania) reference management software, and duplicates were removed. Two independent reviewers screened the titles and abstracts of the retrieved articles for eligibility based on the inclusion and exclusion criteria. Discrepancies between reviewers were resolved through discussion or consultation with a third reviewer. Additionally, full-text articles were obtained for studies that met the initial screening criteria. The same two reviewers independently assessed the full-text articles for final inclusion. The study selection process is documented in Figure 1.

Data Extraction and Management

Data extraction and management were conducted using a standardized form to capture essential information from the included studies. The extracted data encompassed study characteristics, including author(s), year of publication, and study design; focus areas, detailing specific precision medicine applications in emergency care settings; and key findings, highlighting the application of precision medicine principles, impacts on clinical outcomes, and identified barriers and facilitators to implementation. Two reviewers independently performed the data extraction, with any discrepancies resolved through discussion or consultation with a third reviewer to ensure accuracy and consistency.

Quality Assessment

The quality of the included studies was assessed through a general appraisal of their relevance, credibility, and contribution to the topic of PEM. Given the diversity of study designs, including original research articles, reviews, an editorial, and a consensus conference report, and the exploratory nature of this review, a formal quality appraisal using standardized tools was supplemented with a detailed bias assessment. Each study was evaluated based on its relevance to the research question, ensuring that it directly addressed the application of precision medicine principles in emergency care settings.

To provide a transparent evaluation of study quality, we examined potential biases specific to each study type, categorizing risks as low, high, or unclear. Table 1 presents a summary of the risk of bias across the included studies, focusing on factors such as selection, performance, detection, reporting, confounding, and conflict of interest biases. Additionally, the clarity and transparency of objectives, methodologies, and findings were considered to determine the reliability of the information presented. Finally, the contribution of each study to the field was assessed by examining the significance of its findings in advancing the understanding of PEM.

**Table 1:** Summary of the bias assessment across the included studies.

Study	Selection Bias	Performance Bias	Detection Bias	Reporting Bias	Confounding Bias	Conflict of Interest Bias
Lee et al. (2021) [4]	Low	High	High	High	Low	Low
Kingsmore et al. (2015) [5]	High	Low	Low	Low	High	High
Kessler et al. (2017) [6]	High	Low	Low	High	High	Low
Strehlow et al. (2024a) [7]	High	Low	Low	High	High	Low
Limkakeng et al. (2016) [8]	High	Low	Low	High	High	Low
Sanz-García et al. (2024) [11]	High	High	High	High	High	High
Salhi et al. (2024) [12]	Low	Low	Low	Low	Low	Low
Chan et al. (2024) [13]	High	Low	Low	High	High	Low
Strehlow et al. (2024b) [14]	High	Low	Low	High	High	High
Shaban et al. (2024) [15]	Low	Low	Low	Low	Low	Low

Data Synthesis

A qualitative synthesis of the findings was conducted due to the heterogeneity of the studies in terms of interventions and outcomes measured. The studies were grouped based on the focus area of precision medicine applications in emergency care, such as genomic applications, ML, point-of-care diagnostics, and education.

Results

The initial database search yielded 218 records. After removing duplicates and screening titles and abstracts, 35 articles were selected for full-text review. Of these, 25 articles were excluded for not meeting the inclusion criteria, such as not focusing directly on PEM or lacking relevant data. Ultimately, 10 studies were included in the systematic review. The 10 included studies comprised original research articles (n = 5), reviews (n = 3), an editorial (n = 1), and a consensus conference report (n = 1). The studies were published between 2015 and 2024. The characteristics of the included studies are summarized in Table 2.

**Table 2:** Characteristics of the included studies. EM: Emergency Medicine; ML: Machine Learning; PM: Precision Medicine; POCUS: Point-of-Care Ultrasound; PEM: Precision Emergency Medicine; AAA: Abdominal Aortic Aneurysm; ED: Emergency Department

Study	Type	Focus Area	Key Findings
Lee et al. (2021) [4]	Review	Machine learning and precision medicine in EM	Discussed ML and PM applications in EM, potential advantages, and limitations.
Kingsmore et al. (2015) [5]	Original Research	Emergency medical genomes	Demonstrated rapid genome sequencing in critically ill infants for precise diagnoses.
Kessler et al. (2017) [6]	Review	Point-of-care ultrasound in pediatric EM	Discussed POCUS as a tool for personalized pediatric emergency care.
Strehlow et al. (2024a) [7]	Review	Conceptualization of PEM	Defined PEM and identified key drivers and challenges.
Limkakeng et al. (2016) [8]	Review	Systematic molecular phenotyping	Explored molecular phenotyping as a pathway to PEM.
Sanz-García et al. (2024) [11]	Editorial	Biomarkers and early warning scores	Highlighted the need for high-precision EM using biomarkers and scoring systems.
Salhi et al. (2024) [12]	Framework Development and Research Priorities	PEM in healthcare delivery and access	Developed a framework for PEM implementation and identified research priorities.
Chan et al. (2024) [13]	Research Agenda	Precision medicine in EM education	Proposed a research agenda for integrating PM into EM education.
Strehlow et al. (2024b) [14]	Consensus Conference Report	PEM research agenda	Developed a policy-relevant, patient-centered research agenda for PEM.
Shaban et al. (2024) [15]	Systematic Review and Meta-analysis	POCUS in AAA diagnosis	Evaluated the efficacy of POCUS in diagnosing abdominal aortic aneurysm in the ED.

Application of Precision Medicine Principles in Emergency Care

Genomic and molecular applications: Several studies highlighted the use of genomic data in emergency settings. Limkakeng et al. (2016) described how systematic molecular phenotyping, including genomics, proteomics, and metabolomics, can transform emergency care by enabling precise diagnoses based on individual patient profiles [8]. They emphasized that while this approach holds great promise, challenges such as cost, turnaround time, and the need for rapid interpretation of complex data in acute settings are significant barriers that need to be addressed.

ML and data science: Lee et al. (2021) explored the role of ML and artificial intelligence (AI) in EM, discussing how these technologies enhance precision medicine by enabling predictive analytics and personalized care plans [4]. They highlighted that ML is transforming diagnostic processes, especially in predicting conditions such as sepsis, thus improving real-time clinical decision-making, risk stratification, and personalized treatment.

Salhi et al. (2024) developed a framework for integrating big data and technology to improve healthcare delivery and access in EM [12]. They identified core domains such as personalized ED care, expedited prehospital care, and prediction tools for system readiness, underscoring the importance of data science in advancing PEM.

Point-of-care diagnostics and biomarkers: POCUS was identified as a valuable tool for delivering precision care. Kessler et al. (2017) reviewed cutting-edge applications of POCUS in pediatric emergency care, suggesting that it allows for personalized assessment and intervention [6]. Shaban et al. (2024) conducted a systematic review and meta-analysis demonstrating that POCUS is highly effective in diagnosing abdominal aortic aneurysm (AAA) in the ED, supporting its role in PEM [15].

Both Kessler et al. (2017) and Sanz-García et al. (2024) emphasized the importance of using biomarkers and early warning scores (EWS) to create individualized diagnostic pathways in emergency settings [6,11]. Biomarkers and EWS help identify patients at risk for clinical deterioration, adding specificity to diagnostics and optimizing treatment timing and choice, thereby improving outcomes.

Biomarkers and EWS: Sanz-García et al. (2024) highlighted the need for incorporating biomarkers and EWS to enhance diagnostic accuracy and patient outcomes in acute settings [11]. The use of these tools facilitates high-precision EM by enabling clinicians to identify subtle changes in a patient's condition that may indicate impending deterioration.

Health professions education: The integration of PEM into medical education was addressed by Chan et al. (2024), who emphasized the existing gap in precision medicine education among emergency physicians [13]. They proposed a research agenda for educating future emergency physicians, stressing the importance of comprehensive training programs that include structured education in genomic medicine, ML, and AI. By equipping clinicians with these skills, the EM workforce can be better prepared for future precision medicine applications.

Impact on Clinical Outcomes

While the studies suggest potential improvements in diagnostic accuracy and personalized care, direct evidence of improved clinical outcomes is still emerging. Kingsmore et al. (2015) reported that rapid genomic diagnostics could alter clinical management in critically ill infants, potentially improving outcomes by enabling timely, targeted interventions [5]. Shaban et al. (2024) found that the use of POCUS in diagnosing AAA led to accurate detection and management, which could translate into reduced morbidity and mortality [15].

Barriers and Facilitators to Implementation

Barriers: Common barriers identified across the studies include time constraints, technological limitations, education and training gaps, and ethical and privacy concerns. The acute nature of emergency care limits the time available for comprehensive data analysis [7,8], making it challenging to integrate complex genomic data into fast-paced emergency environments. Technological limitations are evident in the need for rapid and accurate diagnostic tools that can be feasibly implemented in the ED, as the cost and turnaround time for technologies like whole-genome sequencing can be prohibitive [12]. Additionally, there is a noted lack of familiarity among emergency physicians with precision medicine tools and techniques [13], highlighting the need for comprehensive training programs. Ethical and privacy concerns related to genetic testing and data security further complicate the adoption of precision medicine, with issues such as informed consent, potential for genetic discrimination, and data security being significant [4,7].

Facilitators: Facilitators for implementing PEM include advancements in technology, interdisciplinary collaboration, and education initiatives. The development of rapid sequencing technologies and portable diagnostic tools supports the practical application of precision medicine in EDs [5,6]. Interdisciplinary collaboration among data scientists, clinicians, educators, and policymakers is essential for integrating precision tools into EM [12,14], as it helps address technological and logistical challenges. Furthermore, incorporating precision medicine into medical curricula prepares future physicians to utilize these tools effectively [13], with structured training in genomic medicine, ML, and AI being crucial for advancing PEM.

The existing literature on PEM is limited but growing. The studies reviewed provide insights into various aspects of precision medicine in emergency care, including genomic applications, ML, point-of-care diagnostics, biomarkers, and educational needs. While there is enthusiasm for the potential of PEM, significant barriers, such as time constraints, technological limitations, ethical concerns, and training gaps, must be addressed to realize its full impact on patient care.

These findings illustrate the current applications and challenges of PEM. Advances in ML, genomic profiling, point-of-care tools, and education are driving the future of this field. Addressing ethical and logistical barriers is crucial for the full implementation of precision medicine in emergency settings, with the goal of enhancing patient outcomes in real-life clinical settings.

Discussion

This systematic review critically evaluated the existing literature on PEM, focusing on the application of precision medicine principles in emergency care settings, their impact on clinical outcomes, and the barriers and facilitators to implementation. The inclusion of additional in-depth analyses in our results has enriched our understanding of how precision medicine is currently being integrated into EM and the potential it holds for transforming patient care.

The findings demonstrate that precision medicine applications in emergency settings can significantly enhance diagnostic accuracy and personalize patient care. An illustrative example is provided by Kingsmore et al. (2015), who utilized rapid whole-genome sequencing (STATseq) in critically ill infants, resulting in timely and precise diagnoses that significantly influenced clinical management and outcomes [5]. This breakthrough underscores the practical feasibility and clinical impact of integrating genomic profiling into acute care settings.

Similarly, the use of POCUS has been shown to facilitate personalized assessment and intervention. Kessler et al. (2017) and Shaban et al. (2024) demonstrated the efficacy of POCUS in pediatric emergencies and AAA diagnosis, respectively, highlighting how point-of-care diagnostics can contribute to precision care by providing immediate, patient-specific information that guides clinical decisions [6,15].

Another promising development is the incorporation of ML and AI into EM. Lee et al. (2021) discussed how ML algorithms are transforming diagnostic processes, particularly in predicting conditions such as sepsis, thereby improving real-time clinical decision-making and risk stratification [4]. These technologies enable the analysis of large datasets to identify patterns and predict outcomes on an individual level, aligning with the goals of precision medicine.

Despite these advancements, the integration of precision medicine into emergency care is not yet widespread. Several barriers contribute to this gap between potential and practice. Time constraints inherent in emergency care limit the feasibility of complex data analysis and interpretation required for genomic and molecular applications [7,8]. Technological limitations, including the cost and turnaround time of advanced diagnostics such as whole-genome sequencing, further impede adoption [12].

Ethical considerations, particularly regarding genetic testing and data privacy, are significant concerns. The use of genomic data raises questions about informed consent, potential genetic discrimination, and the security of sensitive information [4,16]. These issues necessitate the development of robust ethical guidelines and data protection measures.

Insufficient education and training among emergency physicians is another critical barrier. Chan et al. (2024) emphasized the need for comprehensive training programs to equip clinicians with the necessary skills in genomic medicine, ML, and AI [13]. Without adequate education, clinicians may be hesitant to adopt new technologies or may not utilize them effectively, limiting the potential benefits of precision medicine.

When comparing our findings with previously published work, there is a consensus on both the potential benefits of PEM and the challenges that impede its adoption. Rizos et al. (2019) highlighted that while precision medicine has revolutionized fields like oncology, its application in EM is limited due to the acute nature of care and the need for rapid decision-making [17]. This aligns with the barriers identified in our review and underscores the unique challenges of integrating precision medicine into EM.

The theoretical implications of these findings suggest that PEM could significantly enhance patient outcomes by tailoring interventions to individual needs. Practical applications are vast, ranging from the use of ML algorithms to predict patient deterioration to employing genomic data for precise pharmacotherapy in acute settings [18,19]. As discussed by Sanz-García et al. (2024), the use of biomarkers and EWS further illustrates how precision tools can aid in early detection and intervention, potentially reducing morbidity and mortality [11].

Despite the promise, the path to implementing PEM is fraught with challenges. Addressing these barriers will require a multifaceted approach. First, investment in rapid, cost-effective diagnostic technologies suitable for emergency settings is essential, including the development of portable devices and point-of-care tools that provide immediate results to mitigate time constraints and enhance feasibility. Second, collaboration among clinicians, data scientists, educators, and policymakers is crucial, as such interdisciplinary partnerships can drive innovation, address technological limitations, and develop practical solutions tailored to the emergency care environment [12,14]. Third, integrating precision medicine into medical curricula will prepare current and future emergency physicians to adopt these approaches effectively, with training programs focusing on genomic literacy, data interpretation, and ethical considerations [13]. Fourth, establishing standardized protocols and ethical guidelines is imperative to address concerns related to data privacy, informed consent, and genetic discrimination, necessitating prioritization by policymakers and professional organizations [16]. Lastly, advocacy for policies that support the integration of precision medicine into EM, including funding for research and infrastructure, can facilitate widespread adoption. By pursuing these strategies, the implementation of PEM can overcome existing barriers and realize its potential to transform emergency care.

Main Findings

Our review highlights several key findings. First, the evidence base for PEM is limited but growing, with only 10 studies directly addressing the topic. This underscores a significant gap in research and emphasizes the need for more studies to build a robust evidence base. Second, the application of precision medicine principles in emergency settings shows promise for enhancing diagnostic accuracy and personalizing patient care, potentially leading to better clinical outcomes. Third, significant barriers to implementation have been identified, including time constraints, technological limitations, lack of education and training, and ethical concerns, all of which need to be addressed to facilitate the integration of PEM into clinical practice. Fourth, advancing PEM will require interdisciplinary collaboration and education, necessitating cooperation between clinicians, data scientists, educators, and policymakers, as well as the incorporation of precision medicine into medical education to prepare future emergency physicians. Lastly, the development of standardized protocols and guidelines is crucial for the safe and effective implementation of PEM.

Limitations

This review has limitations. The small number of studies included reflects the limited research available on PEM, which may affect the generalizability of the findings. Additionally, the heterogeneity of the studies in terms of focus areas and methodologies makes it challenging to draw definitive conclusions. Publication bias may also be present, as studies with negative findings are less likely to be published.

Future Directions

To advance PEM, several key directions should be pursued. Large-scale, multicenter studies are needed to assess the effectiveness of precision interventions in emergency settings, providing robust evidence for their impact on patient outcomes. Investment in technological innovations is crucial to developing rapid and accurate diagnostic tools that align with the fast-paced nature of emergency care [7]. Additionally, integrating precision medicine into EM curricula will equip clinicians with the necessary skills to utilize these tools effectively. Establishing ethical guidelines and policies that address data privacy and consent issues is essential to ensuring patient trust and compliance with regulatory standards. Finally, fostering interdisciplinary collaboration will help bridge the gap between technological capabilities and clinical application, enabling a more seamless integration of precision medicine into emergency care [20].

## Conclusions

PEM is an emerging field with the potential to significantly improve patient care in emergency settings. These advanced diagnostic tools, including genomic profiling, ML algorithms, and point-of-care technologies, highlight the significant potential of PEM to transform emergency care. While the current literature is limited, the studies reviewed highlight promising applications and underscore the importance of addressing barriers to implementation. By fostering interdisciplinary collaboration, investing in education and technology, and developing supportive policies and ethical guidelines, the integration of precision medicine into emergency care can be realized, ultimately enhancing patient outcomes in acute settings.
